# Alpha‐Mannosidosis in a 3.5‐Year‐Old Girl: A Case Report

**DOI:** 10.1002/ccr3.73276

**Published:** 2026-08-04

**Authors:** Samuel Bonilla Fornes, Maria Pilar Mendez Perez, Marta Torres Diaz, Maria Eugenia Sanchez Gutierrez, Enrique Galán Gómez

**Affiliations:** ^1^ Department of Pediatrics Hospital Materno Infantil, SES Badajoz Spain; ^2^ Department of Pediatrics, Hospital Materno Infantil, SES, School of Medicine University of Extremadura Badajoz Spain; ^3^ Department of Genetics and Immunology Hospital Universitario de Badajoz Badajoz Spain

**Keywords:** alpha‐mannosidosis, enzyme deficiency, enzyme replacement therapy, prognosis, quality of life

## Abstract

Alpha‐mannosidosis is a rare lysosomal storage disease caused by a deficiency of the enzyme alpha‐mannosidase. It manifests as a continuous spectrum of signs and symptoms characterized by dysmorphic features, skeletal abnormalities, delayed psychomotor and speech development, impaired hearing, and psychiatric involvement. When suspected, alpha‐mannosidosis must be confirmed by biochemical and molecular testing, namely, assessment of blood levels of alpha‐mannosidase in leukocytes or fibroblasts and Sanger or next‐generation sequencing of the *MAN2B1* gene. The disease must be diagnosed and treatment started as quickly as possible, since the long‐term prognosis for untreated patients is very poor. Enzyme replacement therapy (ERT, human recombinant alpha‐mannosidase) has replaced allogeneic stem cell transplant as the mainstay of therapy, thus improving disease‐related outcomes with, for example, reduced serum oligosaccharide levels, greater functional capacity, and improved quality of life, all with a good safety profile. We report the seventh case of alpha‐mannosidosis in Spain. The patient was a 3.5‐year‐old girl assessed in the clinical genetics department for developmental delay and marked dysmorphic features (trigonocephaly, exophthalmos, hypertelorism, and a flat nasal bridge). Radiography revealed shortening and thickening of the long bones, as well as metopic and coronal synostosis. Craniosynostosis was treated with surgery. Assessment of alpha‐mannosidase revealed complete absence of enzymatic activity. Genetic analysis revealed the homozygous pathogenic variant of *MAN2B1*, c.2248C>T, which is associated with alpha‐mannosidosis. ERT is the only currently available pharmacological option for treating children with mild‐to‐moderate alpha‐mannosidosis. Without ERT, patients' quality of life would be impaired, and their prognosis would worsen significantly.

## Key Clinical Message

Alpha‐mannosidosis should be considered in patients with delayed neurodevelopment, hearing loss, and dysmorphic features. Diagnosis is sometimes urgent, and the biochemical study should be accompanied by molecular testing. Enzyme replacement therapy is the only pharmacological option in pediatric alpha‐mannosidosis and has proven safe in young children with a good prognosis.

**Abbreviations**
3MSCT: 3‐min stair‐climb test
6MWT: 6‐min walk test
ERT: enzyme replacement therapy

## 1 Background

Alpha‐mannosidosis (OMIM 248500) is a rare lysosomal storage disease caused by a deficiency in the enzyme alpha‐mannosidase [1, 2, 3, 4, 5] leading to intracellular accumulation of mannose‐rich oligosaccharides, with increased excretion in urine [2, 4, 6] and accumulation of mannosylated glycoproteins in tissue [4].

The disease manifests as a continuous spectrum of signs and symptoms [3, 4, 7, 8] characterized by the presence of dysmorphic features, skeletal abnormalities, delayed psychomotor and speech development, hearing impairment, and psychiatric problems [1, 3, 6, 9, 10].

When alpha‐mannosidosis is suspected, the first step is to perform a qualitative urinalysis of mannose‐rich oligosaccharides using high‐performance liquid chromatography and thin‐layer chromatography [1, 3]. Serum oligosaccharide levels in affected patients exceed 4.4–4.5 μmol/L, although an abnormal finding is not diagnostic [1, 2, 3]. Confirmation is by assessment of blood levels of alpha‐mannosidase activity in leukocytes or fibroblasts and by Sanger or next‐generation sequencing of the *MAN2B1* gene [1, 3].

Recent years have seen the development of a new enzyme replacement therapy (ERT) based on human recombinant alpha‐mannosidase [1, 2, 3, 4, 7, 11, 12]. This approach is now used mainly in mild‐to‐moderate forms of the disease, leading to reduced mannose‐rich oligosaccharide levels, a considerable improvement in motor skills, increased lung capacity, significant increases in immunoglobulin levels, and improved quality of life [4, 5, 12, 13].

Allogeneic stem cell transplantation constitutes an additional option. However, this intervention was associated with morbidity and mortality, as well as with a modest improvement in cognitive ability [14].

We report the case of a patient whose symptoms were indicative of alpha‐mannosidosis. Diagnosis was confirmed based on an appropriate degree of suspicion and biochemical and molecular testing. We report 12 months' treatment follow‐up data. The case we report is the seventh patient diagnosed with alpha‐mannosidosis in Spain.

## 2 Case Presentation

The patient was a girl aged 3.5 years who was assessed in the clinical genetics department for developmental delay and mild dysmorphic features.

### 2.1 Personal and Family History

The patient's parents are first cousins. She has a healthy 2‐year‐old brother.

After comprehensive pregnancy monitoring, the patient was born via normal delivery at 40 weeks of gestational age. Somatometric data at birth were normal. Her birth weight was 3280 g. An umbilical hernia had been present since birth. As for psychomotor development, the patient began to roll over at 5 months and walk at 17 months. Language delay was severe, and she remained incontinent at age 3 years.

### 2.2 Disease History

At age 15 months, the patient began to make repetitive movements of her upper and lower limbs owing to nonepileptic paroxysmal disorder. The electroencephalogram was normal. Evaluation in the physiotherapy department at 24 months revealed pes valgus and pes cavus. The patient was assessed by the ENT department owing to hypertrophic tonsils and bilateral serous otitis media. She was admitted to hospital at 2 years and 11 months with pain and inflammation affecting the left knee. Treatment was with arthrocentesis and antibiotic therapy.

The physical examination showed her weight to be 14.5 kg (P44), height 94 cm (P25), and head circumference 47.5 cm (P7). Examination of the head revealed trigonocephaly, horizontal palpebral fissures, exophthalmos, hypertelorism, as well as a broad, flat, and depressed nasal bridge. She had full everted lips and macroglossia, a hoarse voice, normal pinnae, and remnants of both upper incisors, which had fractured. Examination revealed a normal thorax, distended abdomen, and umbilical hernia. The spleen was palpable, as was the liver (1 cm below the costal margin). Her limbs were slightly short, and she had normal female genitalia.

### 2.3 Additional Testing

The blood work‐up revealed normal values for ammonia, pyruvate, lactate, plasma amino acids, and plasma acylcarnitine.

Abdominal ultrasound revealed slight splenomegaly (4 mm in reference to the 12th left rib), umbilical hernia, and rectus abdominis diastasis.

**FIGURE 1:**
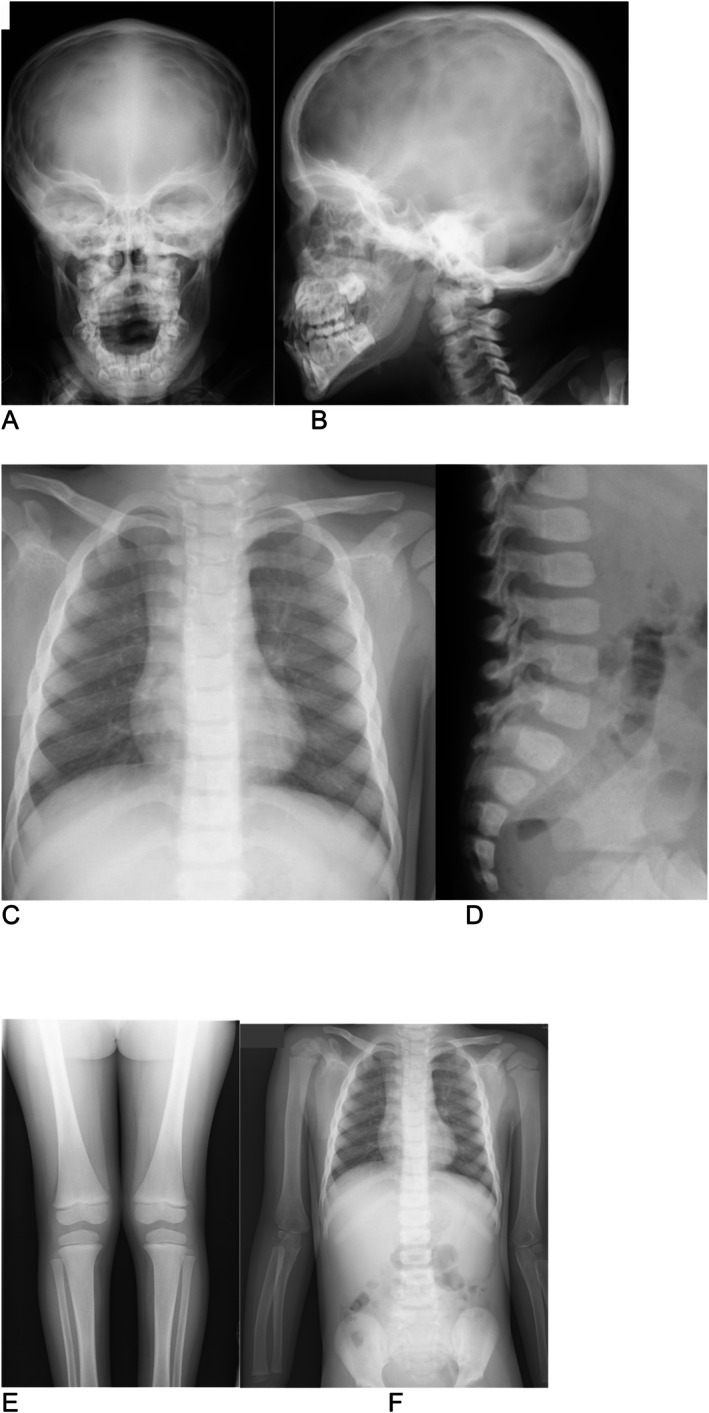
Bone examination in a patient aged 3.5 years. (A, B) Coronal and sagittal images showing copper beaten skull and cranial vault thickening. (C) Thick ribs, sagittal view. (D) Fusion defect on posterior arch of L5. (E, F) Slightly thickened short bones in limbs and widening in metaphyses.

Total skeletal radiography (Figure 1) revealed a thick cranial vault with prominent convolutional markings (copper beaten skull) (Figure 1A,B), thick ribs (Figure 1C), posterior arch fusion defect at L5 (Figure 1D), shortening and thickening of the long bones, and slight metaphyseal widening (Figure 1E,F).

**FIGURE 2:**
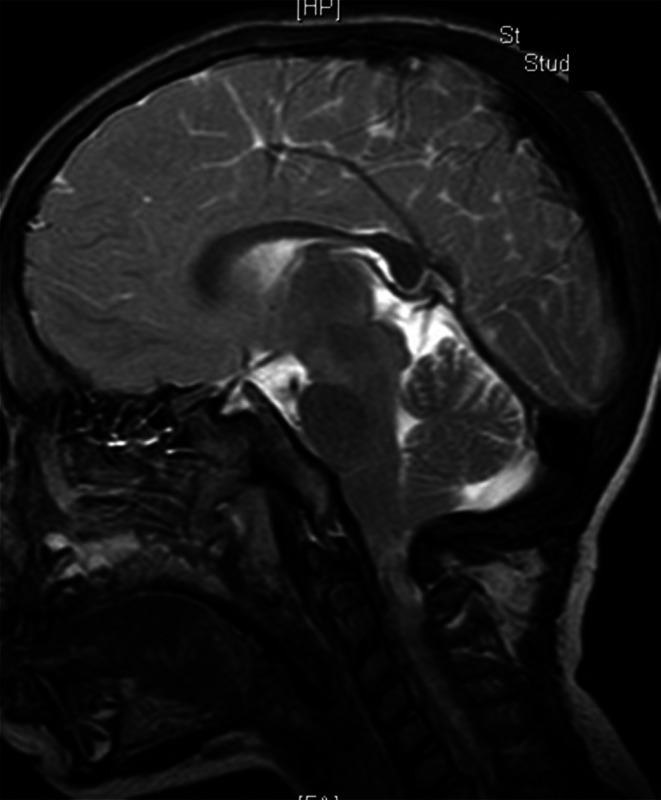
Sagittal view on cranial magnetic resonance imaging. Note dysgenesis of the corpus callosum.

Magnetic resonance imaging of the skull revealed metopic and coronal synostosis and dysgenesis of the corpus callosum (Figure 2).

Trigonocephaly and anterior brachycephaly were treated with surgery. The cardiac evaluation was normal. The ophthalmology evaluation revealed no corneal deposits or crystalline lens opacities, and fundoscopy was normal.

The patient underwent surgery (adenotonsillectomy and bilateral myringotomy with tubes).

Quantitative urinalysis revealed increased excretion of oligosaccharides and normal glycosaminoglycan levels. A study of the enzymatic activity of alpha‐mannosidase revealed the complete absence of the enzyme.

The results of array comparative genomic hybridization were normal.

Targeted exome sequencing (by phenotype) for lysosomal storage disease and skeletal dysplasia yielded the homozygous pathogenic variant of *MAN2B1* c.2248C>T/pHGVS: p.R750W (missense_variant)/Exon 18/24, with autosomal recessive inheritance (NM_000528.3), which is associated with alpha‐mannosidosis. We performed the exome study to assess lysosomal storage disorder and bone dysplasia at the same time as the biochemistry work‐up because the mother was pregnant. The condition had to be diagnosed quickly owing to the potential need for a prenatal genetic study during the mother's current pregnancy.

A genetic study of the parents and sibling showed that they were heterozygous for the variant c.2248C>T (p.R750W) in *MAN2B1*. The mother, who was pregnant, underwent amniocentesis, which revealed homozygous c.2248C>T (p.R750W) of *MAN2B1* in the amniotic fluid. The pregnancy was terminated.

### 2.4 Treatment Initiation and Outcomes

The patient started to receive ERT as an intravenous infusion of velmanase alfa at hospital in June 2022. The starting dose was 1 mg/kg/week, as indicated in the summary of product characteristics [12]. This was well tolerated, and no complications were recorded.

Twelve months after initiation of treatment, the patient remains stable, with no treatment‐related adverse events and good response and tolerability. During this year, the patient's language improved considerably, and speech‐language pathology is also helping, enabling her to develop a wider and more advanced vocabulary and better understanding. Her behavior has also improved, and she now leads a high‐quality normal life. The patient's hearing remains stable.

Before initiation of treatment, the patient's gait was stable, although motor coordination was difficult. In addition, praxis was poor, and she showed little interest in handling objects. After initiation of treatment, her coordination improved (increase in distance covered in the 6‐min walk test [6MWT] and number of steps in the 3‐min stair‐climb test [3MSCT]).

As for hearing impairment, the patient presented normal audiometry parameters in terms of brainstem auditory evoked potentials at the beginning of treatment and after 12 months. Evaluation in the ENT department revealed mucoserous otitis, adenoid hypertrophy, and pediatric obstructive sleep apnea. In April 2022, the adenoids were removed, and drainage tubes were placed in both ears.

## 3 Discussion

Alpha‐mannosidosis is caused by a deficiency in the enzyme alpha‐mannosidase. Inheritance is autosomal recessive [1, 2, 3, 4, 5]. Prevalence is between 1/500,000 and 1/1,000,000 live births [1, 2, 4, 5, 11], with only seven cases reported to date in Spain.

The gene responsible for the disease is *MAN2B1*, for which more than 155 mutations have been identified [1, 3, 6, 11, 15]. *MAN2B1* is located at chromosome 19 (19 p13.2‐q12) [4, 6, 15]. The correlation between genotype and phenotype is still controversial [3, 6].

For years, allogenic stem cell transplant was the only treatment option. However, since 2018, affected patients in Europe have been able to receive ERT based on human recombinant alpha‐mannosidase, which reduces serum oligosaccharide levels, thus increasing catabolism and preventing accumulation in tissue [4, 5, 12, 14]. This approach is indicated for the treatment of non‐neurological manifestations—it has not been shown to cross the blood–brain barrier—in patients with mild‐to‐moderate clinical involvement [12]. Borgwardt et al. [16] also recorded an improvement in cognitive and motor function, with reduced oligosaccharide levels in cerebrospinal fluid, blood, and urine [2, 8, 15].

According to the endpoints evaluated in the clinical development of velmanase alfa, the mean relative change in serum oligosaccharides (S‐oligo) in the study arm was −77.6%. In the case we report, urine oligosaccharide levels were measured at the beginning of the treatment, revealing very high values.

Motor disturbance was evaluated using the 3MSCT and the 6MWT. An improvement in these parameters, especially in children and after long‐term treatment with velmanase alfa, has been reported [4, 5, 14, 15]. We did not observe motor disturbance at the beginning of treatment because of the patient's young age, although the condition stabilized and even slightly improved after 12 months of treatment (Table 1).

**TABLE 1:** 6‐min walk test (6MWT) and 3‐min stair‐climb test (3MSCT) from baseline to the last visit.

	Baseline, age 6 years, 9 months	June 2023
6MWT	343.2 m	396 m
3MSCT	240 steps	254 steps

The main symptoms in younger patients are impaired hearing, recurrent otitis, and early language delay (as in the case we report), with wide variability in symptoms, ranging from completely nonverbal patients to patients with clear speech that is inappropriate for their age [10]. Cases of dysarthria have also been reported [1, 3, 6]. Patients rarely exhibit symptoms at birth [1, 3, 7], thus hampering early diagnosis. In the present case, and even though the diagnostic delay was short (3.5 years), it was necessary to monitor symptoms before alpha‐mannosidosis was suspected.

Impaired language development is characterized by limited vocabulary, poorly intelligible speech, and inappropriate pronunciation [6], as observed at diagnosis in the present case. After 12 months of ERT, the patient's language ability had improved considerably, with a much larger vocabulary. At 6.5 years (after 18 months with ERT), she was able to construct simple 2‐ to 3‐word sentences and could understand instructions when given in context.

Periodic behavioral abnormalities and the presence of psychiatric symptoms (e.g., anxiety, depression, hallucinations, and delirium) have been detected in 70% of patients aged 10–30 years [6, 9]. Given the young age of the patient, psychiatric symptoms were not present, although we did detect behavioral deficiencies that were reversed after 12 months of treatment. The patient showed signs of global developmental behavioral disorder (e.g., an intermittent inappropriate response to her name, poor interaction in play and social contact, and episodes of inappropriate irritability), although these improved after 1 year of treatment. This finding is in line with the results of a recent study on velmanase alfa in children aged < 6 years, in which quality of life was assessed by administering the PEDI questionnaire to the parents. After at least 24 months of treatment with velmanase alfa, raw and scaled scores improved in all children in all domains compared with baseline [17]. Furthermore, during the clinical development of the drug, quality of life improved, as shown by the EuroQol 5 Dimension‐5 Level Questionnaire administered after 48 months of treatment, with absolute change values for the pediatric group of 0.083 (0.136) that exceeded the minimal clinically important difference [18].

Patients with alpha‐mannosidosis have macrocephaly and coarse facial features, such as prominent forehead, broad and rounded eyebrows, flat nasal bridge, macroglossia, and widely spaced teeth, as well as prognathism and short neck [1]. In line with the red flags pointing to a lysosomal disorder, coarse facial features were present in the case we report (see above). Lysosomal storage disorders can present with a broad clinical spectrum, including attenuated phenotypes that are frequently overlooked or diagnosed late [10].

Osteo‐skeletal abnormalities in alpha‐mannosidosis include multiple dysostoses, genu valgum, kyphosis, scoliosis, asymptomatic osteopenia, sclerotic bone lesions, sternal deformities, hip dysplasia, cranial thickening, and malformed vertebrae [1, 6]. In the present case, the main skeletal abnormalities were shortening of long bones and slight metaphyseal widening of the metaphysis (Figure 1E,F).

Alterations of the immune system have been reported, mainly in the form of respiratory and gastrointestinal infections, especially during the first decade of life [1, 6, 7, 8].

The central nervous system abnormalities reported include occipital white matter lesions with local or diffuse signal hyperintensity on MRI, cerebellar or cortical atrophy, and thinning of the corpus callosum [1, 9]. This finding was observed in the MRI scan performed in the present report, which revealed dysgenesis of the corpus callosum. Delayed myelination and hydrocephaly have also been reported [1].

The patient presented with metopic and coronal craniosynostosis. She underwent surgery and progressed well thereafter. Three cases with craniosynostosis have been reported. Craniosynostosis may be a sign of alpha‐mannosidosis and can be considered an extension of the clinical spectrum [3, 8, 19].

Our patient had an umbilical hernia. This condition is observed in a variable number of patients [19].

Despite the diagnostic difficulties, it is very important to diagnose the disease early, given the increasing evidence that the response to treatment is better in children than in adults, which reinforces the importance of early diagnosis of this disease and treatment with replacement therapy [1, 3, 4, 8, 17]. The long‐term prognosis for untreated patients is very poor, with progression of cognitive, skeletal, and neuromuscular impairment over decades. Most patients need a wheelchair for basic mobility and assistance with their activities of daily living [1, 8]. The decrease in life expectancy is determined by neurological involvement and recurrent infection [7].

In order to facilitate early diagnosis of alpha‐mannosidosis, the pediatrician should take the condition into account in the case of a child with delayed neurodevelopment and dysmorphic features (usually less obvious than in patients with mucopolysaccharidosis). Therefore, it is important to publish reports of cases of alpha‐mannosidosis in pediatrics journals.

Since recently, ERT with velmanase alfa has been funded by the public health system in Spain, thus improving access to the drug. Therefore, it is very important to highlight that this is the only medication available to treat affected children. Without treatment, patients' quality of life would be considerably impaired, and prognosis would worsen significantly [6, 12].

## 4 Conclusions

We report a case of alpha‐mannosidosis in which diagnosis was confirmed based on an appropriate degree of suspicion and biochemical and molecular testing. This is the seventh such case in Spain.

The spectrum of signs and symptoms of alpha‐mannosidosis should include craniosynostosis.

In order to facilitate the diagnosis of alpha‐mannosidosis, the pediatrician should take the condition into account when treating patients with delayed neurodevelopment and dysmorphic features.

Diagnosis is sometimes urgent (especially when the mother is pregnant and prenatal testing is necessary). Therefore, molecular testing should be performed together with or before the biochemical study.

Treatment with ERT is safe in young children with a good prognosis.

## Author Contributions

**Enrique Galán Gómez:** conceptualization, supervision, validation, visualization, writing – original draft, writing – review and editing. **Samuel Bonilla Fornes:** data curation, formal analysis, investigation, resources. **Maria Pilar Mendez Perez:** data curation, formal analysis, funding acquisition, investigation, methodology, project administration, resources, software. **Maria Eugenia Sanchez Gutierrez:** formal analysis, investigation, methodology, resources. **Marta Torres Diaz:** data curation, formal analysis, funding acquisition, investigation, methodology, project administration, resources, software.

## Funding

Medical writing for this work was supported by Chiesi Spain S.A.U. including coverage of all publication fees. This funding did not interfere with the study design, data collection, analysis and interpretation of data, the preparation of this article, or the decision to submit it for publication.

## Ethics Statement

The patient's parents gave their consent for the patient to participate in the study and for her medical data and images to be used for publication.

## Consent

The patient's parents gave their written informed consent for the patient's medical data and images to be reported in the present article.

## Conflicts of Interest

The authors declare no conflicts of interest.

## Data Availability

The datasets generated and/or analyzed during the current study are not publicly available because they are from the patient's medical records but are available from the corresponding authors on reasonable request.

## References

[1] N. Guffon , A. Tylki‐Szymanska , L. Borgwardt , et al., “Recognition of Alpha‐Mannosidosis in Paediatric and Adult Patients: Presentation of a Diagnostic Algorithm From an International Working Group,” Molecular Genetics and Metabolism 126, no. 4 (2019): 470–474, 10.1016/j.ymgme.2019.01.024. 30792122

[2] M. Zielonka , S. F. Garbade , S. Kölker , G. F. Hoffmann , and M. Ries , “Ultra‐Orphan Lysosomal Storage Diseases: A Cross‐Sectional Quantitative Analysis of the Natural History of Alpha‐Mannosidosis,” Journal of Inherited Metabolic Disease 42, no. 5 (2019): 975–983, 10.1002/jimd.12138. 31222755

[3] D. Lehalle , R. Colombo , M. O'Grady , et al., “Hearing Impairment as an Early Sign of Alpha‐Mannosidosis in Children With a Mild Phenotype: Report of Seven New Cases,” American Journal of Medical Genetics, Part A 179, no. 9 (2019): 1756–1763, 10.1002/ajmg.a.61273. 31241255

[4] P. Harmatz , F. Cattaeo , D. Ardigò , et al., “Enzyme Replacement Therapy With Velmanase Alfa (Human Recombinant Alpha‐Mannosidase): Novel Global Treatment Response Model and Outcomes in Patients With Alpha‐Mannosidosis,” Molecular Genetics and Metabolism 124, no. 2 (2008): 152–160, 10.1016/j.ymgme.2018.04.003. 29716835

[5] A. M. Lund , L. Borgwardt , F. Cattaneo , et al., “Comprehensive Long‐Term Efficacy and Safety of Recombinant Human Alpha‐Mannosidase (Velmanase Alfa) Treatment in Patients With Alpha‐Mannosidosis,” Journal of Inherited Metabolic Disease 41, no. 6 (2018): 1225–1233, 10.1007/s10545-018-0175-2. 29725868 PMC6326957

[6] D. Malm , H. M. F. R. Stensland , Ø. Edvardsen , and Ø. Nilssen , “The Natural Course and Complications of Alpha‐Mannosidosis—A Retrospective and Descriptive Study,” Journal of Inherited Metabolic Disease 37, no. 1 (2014): 79–82, 10.1007/s10545-013-9622-2. 23739775

[7] V. Nir , L. Bentur , G. Tal , et al., “Comprehensive Cardiopulmonary Assessment in α Mannosidosis,” Pediatric Pulmonology 55, no. 9 (2020): 2348–2353, 10.1002/ppul.24864. 32445542

[8] E. Verrecchia , L. L. Sicignano , M. Massaro , et al., “Caregivers' and Physicians' Perspectives on Alpha‐Mannosidosis: A Report From Italy,” Advances in Therapy 38, no. 1 (2021): 1–10, 10.1007/s12325-020-01574-w. 33231860

[9] J. Majovska , I. Nestrasil , A. Paulson , et al., “White Matter Alteration and Cerebellar Atrophy Are Hallmarks of Brain MRI in Alpha‐Mannosidosis,” Molecular Genetics and Metabolism 132, no. 3 (2021): 189–197, 10.1016/j.ymgme.2020.11.008. 33317989

[10] E. Urizar , E. P. McCarron , C. Gadepalli , et al., “Genetic Insights and Diagnostic Challenges in Highly Attenuated Lysosomal Storage Disorders,” Genes 16, no. 8 (2025): 915, 10.3390/genes16080915. 40869962 PMC12385809

[11] T. Wiesinger , M. Schwarz , T. P. Mechtler , S. Liebmann‐Reindl , B. Streubel , and D. C. Kasper , “α‐Mannosidosis—An Underdiagnosed Lysosomal Storage Disease in Individuals With an ‘MPS‐Like’ Phenotype,” Molecular Genetics and Metabolism 130, no. 2 (2020): 149–152. 32331969 10.1016/j.ymgme.2020.04.001

[12] European Medicines Agency (EMA) , “European Public Assessment Report Lamzede,” 2018, https://www.ema.europa.eu/en/documents/assessment‐report/lamzede‐epar‐public‐assessment‐report_en.pdf.

[13] L. Borgwardt , N. Guffon , Y. Amraoui , et al., “Efficacy and Safety of Velmanase Alfa in the Treatment of Patients With Alpha‐Mannosidosis: Results From the Core and Extension Phase Analysis of a Phase III Multicentre, Double‐Blind, Randomised, Placebo‐Controlled Trial,” Journal of Inherited Metabolic Disease 41, no. 6 (2018): 1215–1223. 29846843 10.1007/s10545-018-0185-0 PMC6326984

[14] M. Mynarek , J. Tolar , M. H. Albert , et al., “Allogeneic Hematopoietic SCT for Alpha‐Mannidosis: An Analysis of 17 Patients,” Bone Marrow Transplantation 47, no. 3 (2021): 352–359, 10.1038/bmt.2011.99. 21552297

[15] J. B. Hennermann , N. Guffon , F. Cattaneo , et al., “The SPARKLE Registry: Protocol for an International Prospective Cohort Study in Patients With Alpha‐Mannosidosis,” Orphanet Journal of Rare Diseases 15, no. 1 (2020): 271, 10.1186/s13023-020-01549-8. 32993743 PMC7525940

[16] L. Borgwardt , C. I. Dali , J. Fogh , et al., “Enzyme Replacement Therapy for Alpha‐Mannosidosis: 12 Months Follow‐Up of a Single Centre, Randomised, Multiple Dose Study,” Journal of Inherited Metabolic Disease 36, no. 6 (2013): 1015–1024. 23494656 10.1007/s10545-013-9595-1

[17] N. Guffon , V. Konstantopoulou , J. B. Hennermann , et al., “Long‐Term Safety and Efficacy of Velmanase Alfa Treatment in Children Under 6 Years of Age With Alpha‐Mannosidosis: A Phase 2, Open Label, Multicenter Study,” Journal of Inherited Metabolic Disease 6, no. 4 (2023): 705–719, 10.1002/jimd.12602. 36849760

[18] L. Borgwardt , N. Guffon , Y. Amraoui , et al., “Health Related Quality of Life, Disability, and Pain in Alpha Mannosidosis: Long‐Term Data of Enzyme Replacement Therapy With Velmanase Alfa (Human Recombinant Alpha Mannosidase),” Journal of Inborn Errors of Metabolism & Screening 6 (2018): e180004, 10.1177/2326409818796854.

[19] P. Lipiński , A. Rózdzynska‐Swiątkowska , K. Iwanicka‐Pronicka , B. Perkowska , P. Pokora , and A. Tylki‐Szymańska , “Long‐Term Outcome of Patients With Alpha‐Mannosidosis—A Single Center Study,” Molecular Genetics and Metabolism Reports 30 (2021): 100826, 10.1016/j.ymgmr.2021.100826. 35242565 PMC8856903

